# Carotid Endarterectomy Versus Carotid Artery Stenting: Survey of the Quality, Readability, and Treatment Preference of Carotid Artery Disease Websites

**DOI:** 10.2196/23519

**Published:** 2020-11-03

**Authors:** Shira Strauss, Michael Yacob, Apoorva Bhandari, Prasad Jetty

**Affiliations:** 1 Division of Vascular Surgery University of Ottawa Ottawa, ON Canada; 2 Division of Vascular Surgery Queen's University Kingston, ON Canada

**Keywords:** patient information, carotid artery disease, carotid endarterectomy, carotid stenting, carotid stenosis, carotid surgery, Google, quality, readability, treatment, preference, online health information

## Abstract

**Background:**

The internet is becoming increasingly more important in the new era of patient self-education. Carotid endarterectomy (CEA) and carotid artery stenting (CAS) are recognized interventions to treat patients with carotid artery stenosis. Using the Google search platform, patients encounter many websites with conflicting information, which are sometimes difficult to understand. This lack of accessibility creates uncertainty or bias toward interventions for carotid artery disease. The quality, readability, and treatment preference of carotid artery disease (CAD) websites have not yet been evaluated.

**Objective:**

This study aimed to explore the quality, readability, and treatment preference of CAD websites.

**Methods:**

We searched Google Canada for 10 CAD-related keywords. Returned links were assessed for publication date, medical specialty and industry affiliation, presence of randomized controlled trial data, differentiation by symptomatic status, and favored treatment. Website quality and readability were rated by the DISCERN instrument and Gunning Fog Index.

**Results:**

We identified 54 unique sites: 18 (33.3%) by medical societies or individual physicians, 11 (20.4%) by government organizations, 9 (16.7%) by laypersons, and 1 (1.9%) that was industry-sponsored. Of these sites, 26 (48.1%) distinguished symptomatic from asymptomatic CAD. A majority of sites overall (57.4%) and vascular-affiliated (72.7%) favored CEA. In contrast, radiology- and cardiology-affiliated sites demonstrated the highest proportion of sites favoring CAS, though they were equally likely to favor CEA. A large proportion (21/54, 38.9%) of sites received poor quality ratings (total DISCERN score <48), and the majority (41/54, 75.9%) required a reading level greater than a high school senior.

**Conclusions:**

CAD websites are often produced by government organizations, medical societies, or physicians, especially vascular surgeons. Sites ranged in quality, readability, and differentiation by symptomatic status. Google searches of CAD-related terms are more likely to yield sites favoring CEA. Future research should determine the extent of website influence on CAD patients’ treatment decisions.

## Introduction

The internet is a popular source of information for Canadians seeking medical advice. According to Statistics Canada, Internet User Surveys revealed that 91.3% of Canadians used the internet [1], and 69.9% of home internet users searched for health information online [2]. With this trend toward health information acquisition online, carotid artery disease (CAD) poses a particular challenge for medical websites because there is no consensus on its optimal management [3,4]. Mixed interpretation of data, rapid evolution in technology and expertise, and pharmacotherapy improvements have led to inconsistent treatment guidelines and practice patterns across specialties and organizations [5]. Consequently, CAD patients searching for ways to treat their condition online may struggle to find a clear answer.

The Carotid Revascularization Endarterectomy versus Stenting Trial (CREST) aimed to settle the carotid endarterectomy (CEA) versus carotid artery stenting (CAS) debate by eliminating confounding factors present in earlier CAD clinical trials. Strict CAS operator training requirements were implemented, standardized embolic protection devices were employed, and cardiac enzymes, electrocardiogram changes, and clinical presentations were routinely monitored. With its publication in 2010, CREST revealed no significant difference between CAS and CEA up to four years for the composite primary outcome: periprocedural stroke, myocardial infarction (MI), or death and subsequent ipsilateral stroke. The CEA group did demonstrate higher rates of MI and the CAS group higher rates of stroke. [6]. Ten-year CREST results published in March of 2016 yielded similar results that were sustained when the endpoints were stratified by symptomatic status, age, sex, or degree of stenosis [7]. Translation of CREST trial results into clinical practice was explored in a recent study by Otite et al [8]. Interestingly, the utilization of CAS increased post-CREST (2011-2014) compared with pre-CREST (2007-2010) for patients >70 years of age in the United States. Similarly, Hussain et al investigated the effects of clinical trial publications, including the CREST trial, on rates of carotid revascularization procedures in Ontario, Canada, between 2002-2014 [9]. In this period, CEA utilization decreased by 36%, while CAS increased by 72%. The CREST trial and the publication of other conflicting trials between 2006-2010 were associated with a decline in CEA rates [9]. The contradiction in clinical practice and trial results may be attributed to an interplay of multiple factors including, but not restricted to, differential interpretation of trial results, advances in CAS technology, availability and accessibility of physician providers and physician specialty.

CEA has traditionally been the treatment of choice for patients with severe and/or symptomatic carotid stenosis. Nevertheless, with continued advances in best medical therapy (BMT) and the recent equivalent long-term CREST results, BMT and CAS challenge CEA as primary treatment modalities [10]. Variation in patient anatomy, age, comorbidities, surgical risks, use of embolization protection devices, and operator experience further complicate treatment decisions for individual patients. Since many patients today look to the internet for medical advice, we sought to identify the most easily accessible websites to patients, evaluate their quality, readability, and balance of information, and determine whether there was a preference for one treatment option.

## Methods

### Site Selection

To generate a list of easily accessible CAD websites, we searched ten keywords commonly associated with CAD, such as “carotid stenosis,” “carotid stenting,” and “carotid endarterectomy” in Google Canada (Multimedia Appendix 1). Google searches yield approximately 10 unique links per page; the top ten websites returned by each search were recorded for a total of 100 websites. Of this sample, websites were excluded from content evaluation if they were repeats from a previous search. Repeat links were recorded to track how frequently a particular website appeared in similar searches. Of all the websites consulted, none was found to be a broken link, lack information regarding carotid disease, require a paid subscription, or be inaccessible.

### Demographic Information

Demographic information collected from each site included (1) type of organization that created the website (specifically: physician/hospital/medical department, government organization, industry, layperson, other), (2) specialty affiliation of the website or its authors, (3) year of publication, (4) inclusion of randomized control data, and (5) differentiation between symptomatic and asymptomatic carotid disease for treatment preference.

### Assessment Tools

#### Gunning Fog Index

Each site was evaluated for readability using the Gunning Fog Index (GFI). The GFI estimates the years of formal education required to understand a passage based on the passage’s average sentence length and the number of complex words. For ease of use and avoidance of human error, an online calculator was used to calculate the GFI for each website [11]. If a website was divided into various webpages, we evaluated the summary overview webpage or the webpage that encompassed the bulk of the information contained in that site. Texts with near-universal understanding generally require a GFI score <8, indicative of an eighth-grade reading level; a score >12, equivalent to a high school senior reading level, is considered too difficult for the general populous [12].

#### DISCERN Instrument

Two researchers independently evaluated each site (SS, MY) for reliability and quality using the DISCERN instrument [13]. The DISCERN instrument consists of three sections totaling 16 questions, each with a rating scale from 1 (low, with serious or extensive shortcomings) to 5 (high, with minimal shortcomings). Section one evaluates each publication’s reliability based on various factors, including relevance, sources, and balance. Section two explores the quality of information concerning treatment options available. Section three consists of an overall rating of the publication based on sections one and two. Websites that received total DISCERN scores <48 were considered poor quality, as 48 represents an average score of <3 across each subsection.

We deviated from the company’s instructions for section two (quality of information) in our use of the DISCERN instrument. Rather than evaluate each site according to how it addressed a single treatment choice, we evaluated each website according to how it addressed all three major treatment modalities for carotid stenosis: BMT, CEA, and CAS. Thus, if a website only addressed two out of the three main treatment options, that website automatically lost a minimum of one point for each question concerning the description, benefits, risks, and impact of the treatments. This method provided more consistency when comparing a site’s ability to honestly and thoroughly inform patients about all treatment options available for carotid disease.

### Site Preference

An overall impression of preferred treatment was recorded for each website. Websites were considered to prefer CEA if they (1) stated that CEA was the standard of care; (2) started the discussion with CEA; (3) devoted far lengthier text to CEA without necessarily declaring that it was better; (4) and/or presented CAS as an alternative treatment intended for special circumstances only. These sites often described CEA as “older and effective,” “very safe,” “traditional,” and “durable.” Websites were considered to favor CEA and CAS equally if they devoted equal amounts of text to each treatment option and/or did not imply that one treatment was preferable to the other. Websites were considered to prefer CAS if they emphasized CAS as a newer, promising, less invasive option with a shorter hospital stay or devoted far lengthier text to CAS without necessarily declaring that it was better. Treatment preference was recorded as not applicable in websites that focused on transient ischemic attacks (TIAs) and strokes of various etiologies since these did not deal exclusively with carotid disease. For websites that recommended different therapies based on symptomatic status, preference was determined based on the recommendation for the symptomatic patient of average surgical risk.

### Statistical Analysis

Statistical analysis was performed in Microsoft Excel. Interrater reliability for the DISCERN ratings was assessed using the Spearman correlation coefficient. Total DISCERN scores were averaged between the two evaluators. Differences between average total DISCERN and GFI scores for sites that preferred CEA, sites that preferred CAS, and sites that presented CEA and CAS equally were calculated using analysis of variance testing. Chi-square test of independence or fisher’s exact test as appropriate, was performed using an online calculator to determine whether there was a relationship between higher DISCERN scores and the presence of randomized controlled trial (RCT) data among the websites assessed. Spearman correlation coefficient was also employed to determine whether there was a correlation between DISCERN and GFI scores.

## Results

### Site Demographics

A total of 54 unique CAD websites were identified using the search terms. Among these, 18 (33.3%) were produced by medical societies or individual physicians, 11 (20.4%) were produced by government organizations, 9 (16.7%) were produced by laypersons, and 1 (1.9%) was industry-sponsored (Table 1). Of the websites affiliated with or authored by a particular specialty/specialist, the three most common affiliations were vascular surgery (11 sites), neurology (7 sites), and internal medicine (6 sites) (Table 1). Of note, sites with multiple authors from different specialties were tallied multiple times in this category for each additional specialty represented among the authorship. We found that 44 (81.5%) websites were published after CREST, and 18 (33.3%) mentioned RCT data. Symptomatic was distinguished from asymptomatic disease on 26 sites (48.1%), 14 sites (25.9%) did not distinguish disease types, and 14 sites (25.9%) were excluded from this category because they were symptomatic stroke sites addressing various stroke etiologies (Table 1).

**Table 1:** Website demographics.

Characteristic		Websites
**Organization type, n (%)**		
	Medical—society	8 (14.8)
	Medical—Doctor of Medicine (MD)/Doctor of Osteopathic Medicine (DO)	10 (18.5)
	Government	11 (20.4)
	Medical—hospital/clinic	8 (14.8)
	Medical—journal	4 (7.4)
	Medical—university affiliation	2 (3.7)
	Layperson	9 (16.7)
	Industry	1 (1.9)
	Other	1 (1.9)
**Medical specialty, n**		
	Vascular	11
	Neurology^a^	7
	Neurosurgery	4
	Cardiology^a^	4
	Internal medicine	6
	Family medicine	2
	Emergency medicine	2
	Radiology^a^	2
	Other^b^	3
	Not specified^c^	23
**Time of Publication, n (%)**		
	Pre-CREST^d^	7 (13)
	Post-CREST	44 (81.5)
	Not reported	3 (5.6)
**RCT^e^ data presented, n (%)**		
	Yes	18 (33.3)
	No	36 (66.7)
**Distinguish symptomatic vs asymptomatic, n (%)**		
	Yes	26 (48.1)
	No	14 (25.9)
	Not applicable	14 (25.9)

^a^Neurology includes interventional radiology; cardiology includes interventional cardiology; and radiology includes interventional neuroradiology.

^b^Other includes radiation oncology, physical and rehabilitation medicine, and rheumatology.

^c^Not specified includes MD unspecified, non-MD, and unspecified author.

^d^CREST: Carotid Revascularization Endarterectomy Versus Stenting Trial.

^e^RCT: randomized controlled trial.

### DISCERN and GFI Data

DISCERN scores from researchers SS and MY demonstrated strong interrater reliability (Spearman ρ=0.98). When averaged between the two researchers, DISCERN scores for all sites ranged from 28.5 to 76 out of a possible 80 points. A total of 21/54 websites (38.9%) received a poor-quality rating (total DISCERN score <48). There was no significant difference between average total DISCERN scores for sites that preferred CEA, sites that preferred CAS, and sites that presented CEA and CAS equally (*P*=.85). Sites with the ten highest DISCERN scores were more likely to contain RCT data than the remaining sites (*P*=.012). Specialty affiliation among sites with the top 10 DISCERN scores included neurology/neurosurgery (3 sites), vascular surgery (2 sites), and cardiology (2 sites). The remaining three sites did not state affiliation with any specialty, and two were Wikipedia pages.

GFI readability scores ranged from 7.7 to 29.1, and 13 websites received a GFI score of <12, corresponding with a reading level at or below that of a high school senior. There were no statistically significant differences between average GFI scores for sites that preferred CEA, sites that preferred CAS, and sites that presented CEA and CAS equally (*P=*.99). GFI and DISCERN scores demonstrated a weakly positive correlation (Spearman ρ=0.34), indicating that websites containing a higher quality of information do not necessarily require a higher reading level.

### Treatment Preference

Overall, most websites (31/54, 57%) demonstrated a preference for CEA, 8/54 (15%) presented CEA and CAS as equal treatment modalities, and 8/54 (15%) demonstrated a preference for CAS. Treatment preference was considered not applicable (N/A) in 7/54 (13%) of sites due to focus on stroke and TIA of various etiology rather than carotid disease alone. While recommended by most sites in conjunction with either CEA or CAS, best medical therapy was not cited as the best treatment modality *alone* in cases of average surgical risk with sufficiently severe carotid stenosis to warrant intervention. Among the ten sites with the highest DISCERN scores, CEA was preferred in six, CEA and CAS were presented equally in two, and CAS was preferred in two.

Vascular surgery was the most common specialty affiliation, with 72.7% of vascular-affiliated sites favoring CEA, 9.1% presenting CEA and CAS equally, and 18.2% favoring CAS. Likewise, sites affiliated with neurology, neurosurgery, internal medicine, and family medicine more often demonstrated a preference for CEA (ranging from 50%-100% of websites affiliated with that specialty) than for CAS or no preference (Table 2). Websites affiliated with interventional radiology or cardiology demonstrated the highest proportion of sites favoring CAS, though they were equally likely to favor CEA (Table 2).

**Table 2:** Website treatment preferences by medical specialty of the websites’ authors.

Author’s medical specialty affiliation (N=41)	Treatment preference, n (%)			
	Carotid endarterectomy	No preference	Carotid artery stenting	Not applicable
Vascular surgery (n=11)	8 (72.7)	1 (9.1)	2 (18.2)	0 (0.0)
Neurology (n=7)	6 (85.7)	0 (0.0)	1 (14.3)	0 (0.0)
Cardiology (n=4)	1 (25.0)	1(25.0)	1 (25.0)	1 (25.0)
Neurosurgery (n=4)	4 (100.0)	0 (0.0)	0 (0.0)	0 (0.0)
Internal medicine (n=6)	3 (50.0)	0 (0.0)	2 (33.3)	1 (16.7)
Interventional radiology (n=2)	1 (50.0)	0 (0.0)	1 (50.0)	0 (0.0)
Family medicine (n=2)	2 (100.0)	0 (0.0)	0 (0.0)	0 (0.0)
Emergency (n=2)	0 (0.0)	1 (50.0)	0 (0.0)	1 (50.0)
Other (n=3^a^)	2 (66.7)	0 (0.0)	0 (0.0)	1 (33.3)

^a^Other includes radiation oncology, physical and rehabilitation medicine, and rheumatology.

**Figure 1:**
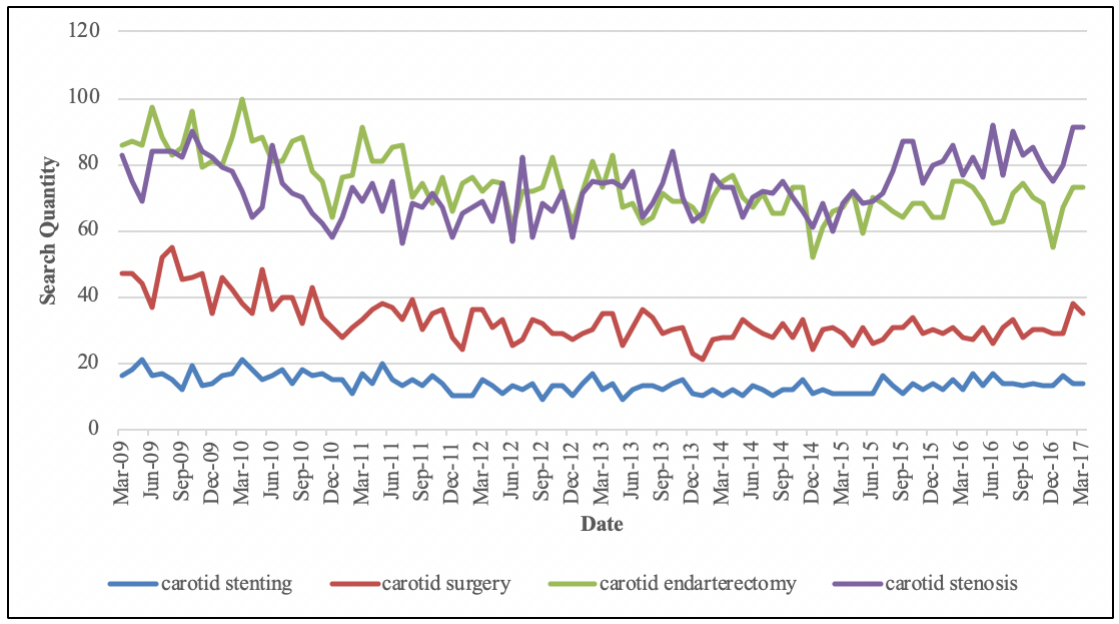
Google search trends by keyword since 2009.

Among the 10 keywords searched, 7 yielded a majority of sites that favored CEA. Only one keyword—“carotid stenting”—yielded a majority of sites that favored CAS (Table 3). Of all 8 sites that demonstrated a preference for CAS, 7 appeared in the “carotid stenting” search, 1 appeared in the “carotid artery stenosis” search, and 1 appeared in the “mini stroke” search. The remaining 7 keywords yielded no sites that favored CAS. Two keywords—“TIA” and “mini stroke”—accounted for all the sites where treatment preference was deemed not applicable. Every keyword searched generated at least one site that presented CEA and CAS as equal treatment modalities (Table 3). Analysis of Google search trends dating back to 2009 revealed that “carotid stenting” was searched less frequently than “carotid endarterectomy” and “carotid surgery” (Figure 1).

**Table 3:** Website treatment preference by keyword search.

Keyword search	Treatment preference (number of websites)			
	Carotid endarterectomy, n	Treatments presented as equivalent, n	Carotid artery stenting, n	No preference, n
Carotid endarterectomy	9	1	0	0
Carotid stenosis	8	2	0	0
Carotid artery stenosis	8	1	1	0
Carotid stenting	2	1	7	0
Carotid surgery	9	1	0	0
Carotid blockage	6	4	0	0
Carotid disease	7	3	0	0
TIA^a^	5	1	0	4
Mini stroke	2	1	1	6
Carotid treatment	4	6	0	0

^a^TIA: transient ischemic attack.

## Discussion

### Principal Findings

High rates of reported information-seeking and use of web-based health technology places an onus on health care providers and educators to capitalize on these resources for disease education and management. Healthcare websites must be accessible, have high usability and reliability, and accommodate the average reading level of American adults, which is reportedly between the seventh- and eighth-grade [14,15]. To our knowledge, this is the first study to explore the quality, readability, and treatment preference of CAD websites. We found that CAD websites were often produced by medical societies or physicians (18/54, 33.3%) and government organizations (11/54, 20.4%). Websites ranged in quality and readability, and higher quality CAD websites did not necessarily require higher user reading levels. Treatment preference varied as a function of physician specialty, with vascular surgery-affiliated websites favoring CEA and interventional radiology and cardiology-affiliated websites favoring CAS.

Consistent with current literature and guidelines, the majority of CAD websites demonstrated a preference for CEA. Abbott et al conducted a systematic review of guideline recommendations for the management of asymptomatic and symptomatic CAD published between 2008 and 2015. Of 28 guidelines with asymptomatic and 33 guidelines with symptomatic CAD procedural recommendations, 24 (86%) and 31 (94%) endorsed CEA for patients with average surgical risk. For symptomatic patients deemed higher surgical risk (due to comorbidities, unfavorable carotid anatomy, etc), CAS was endorsed by 27 guidelines (82%) [5]. Recently, Brott et al conducted a pooled analysis of individual patient-level data acquired from the four largest RCTs completed to date, assessing the relative efficacy of CAS versus CEA for treatment of symptomatic carotid stenosis. They showed that long-term outcomes continue to favor CEA. However, improvements in the periprocedural safety of CAS could provide similar outcomes of the two procedures in the future [16]. The majority of CAD websites reflect the treatment preference of medical practitioners for patients at average surgical risk.

The majority of RCTs investigating CEA versus CAS found significant differences in perioperative outcomes, largely in symptomatic patients [17]. In our study, 48.1% (26/54) of CAD websites distinguished between symptomatic and asymptomatic CAD. Complication risks associated with CAE and CAS are higher in symptomatic than in asymptomatic patients [4,18]. Researchers have speculated that symptomatic carotid disease is associated with greater overall cardiovascular risk. Studies have also shown that annual stroke risk is lower for asymptomatic patients than symptomatic patients [18]. The benefit of CEA is greater among symptomatic compared [19-23] and remains the gold standard for this patient population [24,25]. Currently, clinical equipoise remains regarding the optimal management of asymptomatic CAD, as evidenced by the recent review article by Abbot et al and the response by Cambria et al, with the former advocating for optimal medical intervention as routine practice and the latter defending the use of mechanical intervention for asymptomatic patients [3,4]. Differentiation by symptomatic status is crucial in determining disease management, procedural risk, and subsequent treatment preference.

Treatment preference for CAD websites varied as a function of physician specialty. Variation in patient treatment preferences is largely physician-driven, as the patients often depend on their physicians to prescribe appropriate treatment [26]. Provider enthusiasm for treatment recommendations may be driven by several factors, including availability, accessibility and operator experience, sociodemographic factors, and provider specialty.

Wallaert et al examined the relationship between physician specialty and annual rates of CAS and CEA using Medicare claims from 2002 to 2010 [26]. Cardiologists performed the majority of CAS procedures, and regions with the highest proportion of cardiologists performed the most CAS procedures. Cardiologists and interventionalists have led efforts to extend CAS funding, while surgeons and neurologists have cautioned against expanding CAS approval outside of clinical registries and trials [26]. These findings are consistent with our findings that vascular surgery-affiliated websites favor CEA, and interventional radiology and cardiology-affiliated websites are more likely to favor CAS by comparison. Interestingly, a study by Keogh et al found that the number of available online CAS-related peer-reviewed sources is double the number of hospital- or health service-generated resources; the opposite is true for CEA [27]. Hospital and health service resources lend themselves to patient populations, which is reflected in the observation of higher readability of CEA than CAS resources [27]. The source of information—physician specialty, peer-reviewed sources, hospitals, or health services—influence website treatment preference in addition to readability based on the resource’s intended audience.

It is recommended that patient health materials be written at or below the fourth to sixth-grade reading level by the American Medical Association (AMA), National Institutes of Health, and the Centers for Disease Control and Prevention [28]. If written below the sixth-grade level, material is considered easy to read; if written between the seventh and ninth grade levels, it is of average difficulty; and if written above the ninth grade level, it is difficult to read [14]. A minority of CAD websites included in our study (13/54, 24%) received a GFI score of less than twelve, corresponding with a reading level at or below that of a high school senior. A recent study also found that 99.5% of online cardiovascular disease-related health education materials recommended by the AMA were written above the fifth to sixth-grade level [29]. Our study found that the CAD websites’ readability was higher than recommended, which could have far-reaching implications for patients’ health literacy [29]. However, it is important to note that readability is only one element of literacy. The GFI may not reflect the reading level as it relies on the number of syllables in a word and the number of words in a sentence. Overall readability may be influenced by images, layout, design, and content organization [30]. As the internet is a growing resource for health information, it is critical to ensure web-based health resources are written at a level accessible to the general patient population.

Additional limitations of this study must be taken into account. The literacy level of carotid stenosis patients may differ from that of the average American, and web-based resources written at a higher readability level may or may not be appropriate for this subgroup. While the DISCERN tool has demonstrated validity and reliability for evaluating the quality of online health information for treatment choices across conditions, there is subjectivity involved for certain rating criteria, which introduces interstudy variability when comparing studies and interrater variability within studies. However, our study demonstrated strong interrater reliability (Spearman ρ=0.98). Also, this study looked at static web-based delivery of health education. There is speculation that an interactive health education delivery approach, compared with a static one, may allow health material to be tailored to readers of a wide breadth of educational backgrounds. The literature has shown that adults with chronic illnesses have associated online health information use with behavior changes and decision-making [31]. Future research should assess how interactive web-based technologies, such as blogs and social networking sites, compared to websites, affect patient-provider communication.

This study did not assess the usability and social reach of CAD websites. Future studies should evaluate how websites are engaging audiences. Many methods can be used to do so, including but not limited to (1) the LIDA online app to assess the usability of healthcare websites [27]; (2) global estimated website traffic over 30 days and over 3 months; and (3) counts of social bookmarking/networking links [31]. It is well known that patient preference for participation in health care varies greatly. Future research would also benefit from evaluating CAD patients’ decision-making preferences and preferences for online information regarding treatment options. The Health Information Wants Questionnaire collects data on the information and associated decision-making autonomy patients want in seven areas of health care [32].

### Conclusion

CAD websites were most often affiliated with, or authored by, vascular surgeons, and CAD-related Google search terms were more likely to yield sites favoring CEA. Sites ranged in quality, readability, and differentiation by symptomatic status. Further research is needed to determine if website treatment preferences consistently and appropriately influence final treatment decisions by patients with carotid artery disease.

## Appendix

Multimedia Appendix 1 List of keywords used for Google Search.

## Abbreviations

AMA: American Medical Association
BMT: best medical therapy
CAD: coronary artery disease
CAS: carotid artery stenting
CEA: carotid endarterectomy
CREST: Carotid Revascularization Endarterectomy versus Stenting Trial
GFI: Gunning Fox Index
MI: myocardial infarction
RCT: randomized controlled trial
TIA: transient ischemic attack
