# Supplementary material for: Carotid Endarterectomy Versus Carotid Artery Stenting: Survey of the Quality, Readability, and Treatment Preference of Carotid Artery Disease Websites
Source: Interact J Med Res. 2020 Nov 3;9(4):e23519. doi: 10.2196/23519 (PMC7671836; doi:10.2196/23519)
Supplement: Multimedia Appendix 1 [file ijmr_v9i4e23519_app1.docx]

**Appendix A**

List of ten keywords searched in Google Canada: carotid endarterectomy, carotid stenosis, carotid artery stenosis, carotid stenting, carotid surgery, carotid blockage, carotid disease, transient ischemic attack, mini stroke, and carotid treatment.
